# Supplementary material for: Laser-Induced Intracellular Delivery: Exploiting Gold-Coated Spiky Polymeric Nanoparticles and Gold Nanorods under Near-Infrared Pulses for Single-Cell Nano-Photon-Poration
Source: Micromachines (Basel). 2024 Jan 23;15(2):168. doi: 10.3390/mi15020168 (PMC10890628; doi:10.3390/mi15020168)
Supplement: Supplementary file 1 [file micromachines-15-00168-s001.zip › micromachines-2789147-supplementary.pdf]

Supporting Information

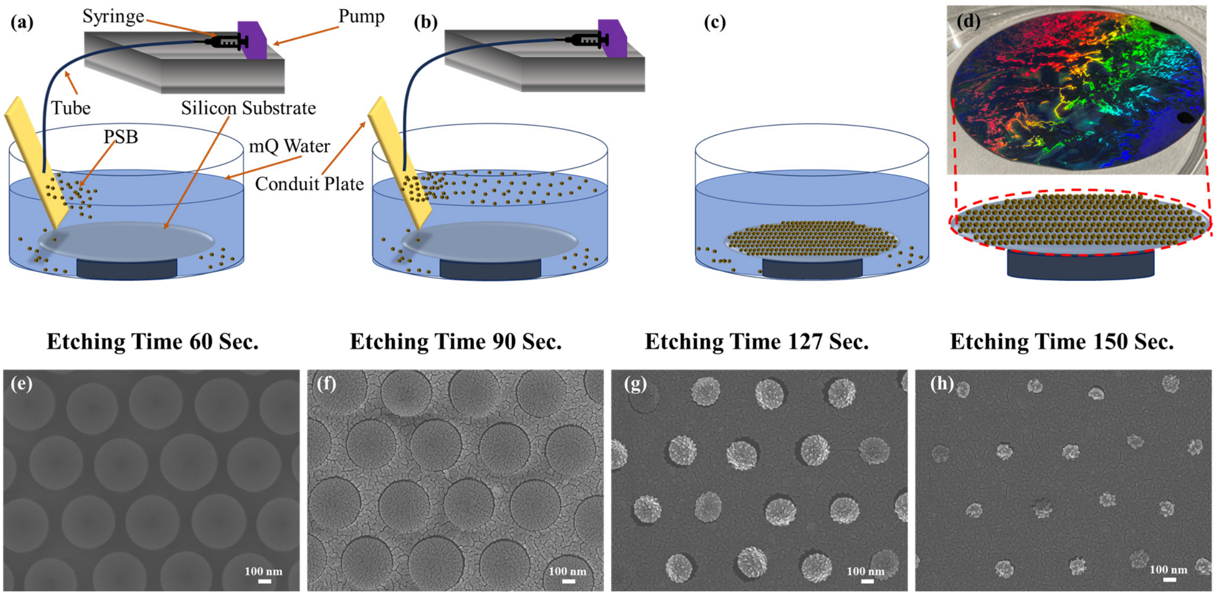

Figure S1. Illustration of the monolayer preparation process and scanning electron microscopy (SEM) images at different reactive ion etching (RIE) parameters. (a) Monodisperse 530 nm polystyrene beads (PSBs) are combined with ethanol and mQ water and meticulously spread onto a water surface using a conduit plate (yellow). (b) PSBs achieved full close-packing on the water surface. (c) The close-packed PSB monolayer is gradually settled onto the wafer surface through a slow and controlled drainage of water. (d) Transfer of the PSB monolayer to a silicon substrate is executed through a gradual water drainage process, revealing a distinct diffraction pattern and an iridescent image [inset]. (e, f, g, h) SEM images depicting hexagonal symmetry with varying etching times of 60, 90, 127, and 150 seconds, respectively.

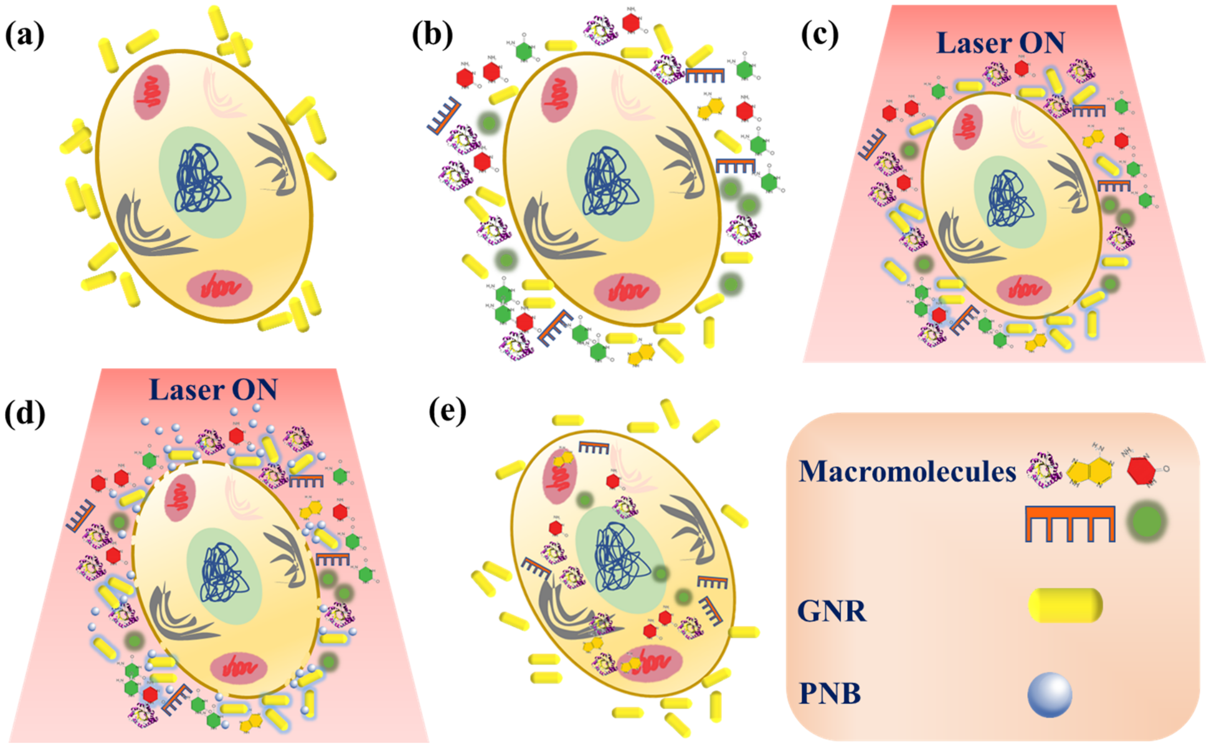

Figure S2. Schematic representation of the GNR mediated photon-portaion mechanism for enhanced delivery of micro/macromolecules (a) GNR-PEG nanoparticles attach to cell membranes after incubation. (b) Unbound GNRs were washed and removed using PBS, and cargo molecules were added before laser exposure. (c) The nanosecond-pulsed laser induces electromagnetic field enhancement near nanoparticle edges and the smooth surface of the GNR. (d) Laser pulses create PNBs at the GNR-cell membrane interface, forming transient pores for molecule delivery. (e) Successful intracellular delivery via transient pores with subsequent membrane resealing and cell viability highlights photon-poration efficacy.

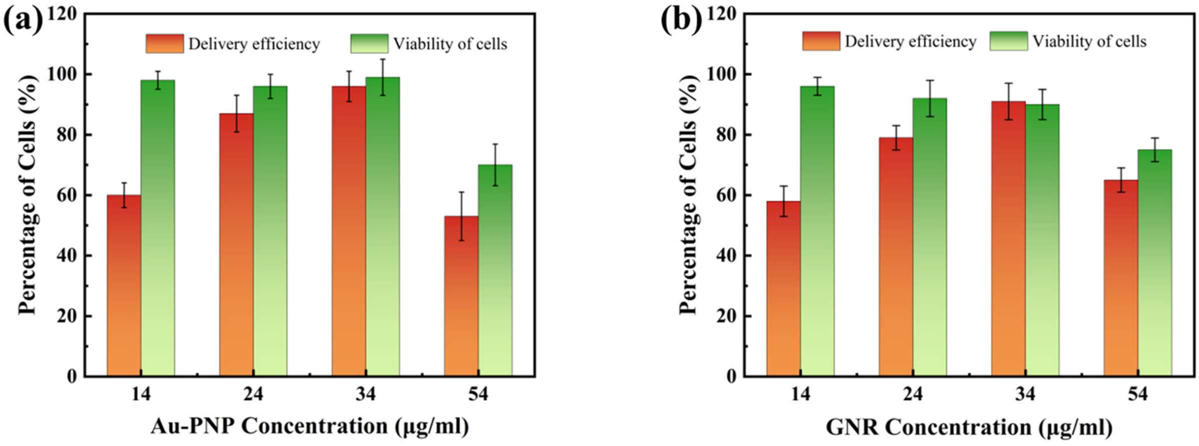

Figure S3. Delivery efficiency and viability of HeLa cells with different Au-PNP and GNR concentrations at 670 nm and 1030 nm respectively. The maximum efficiency and viability were observed at 34 μg/mL. For higher particle concentrations, the delivery efficiency and viability are reduced dramatically. The laser exposure was 30 seconds at a 10 Hz pulsing frequency with 27 mJ/cm^2 laser fluence. Data show average ± standard deviation (n =3 replicate).

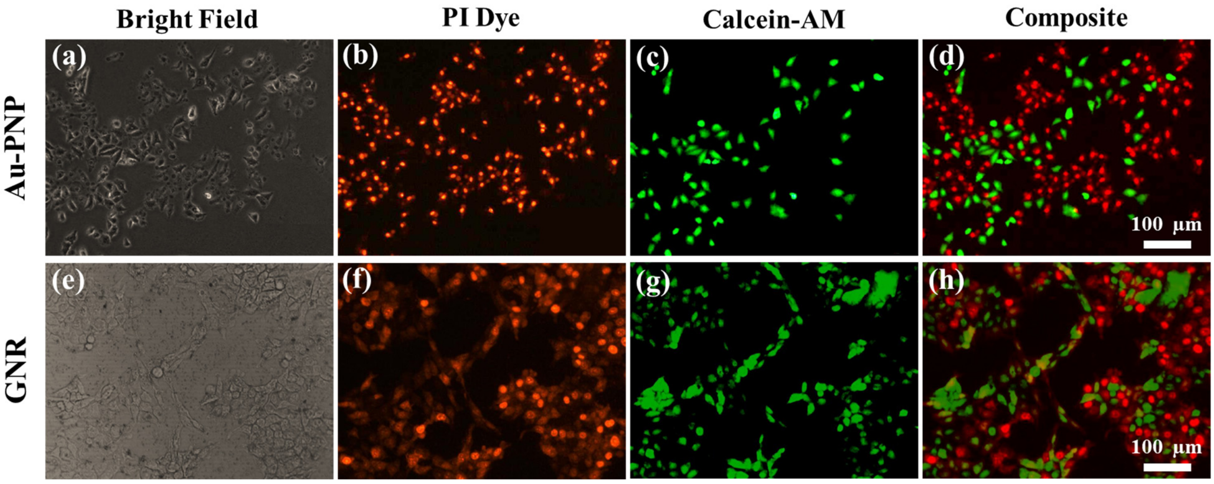

Figure S4. PI dye delivery and viability with a higher concentration of Au-PNP and GNR (54 μg/mL) for HeLa cells (a,e) bright field image after laser exposure area shows debris on top of the cells as well as surrounding the cells (b,f) PI dye delivery (c,g) cell viability test using Calcein-AM (d,h) Composite image of PI dye and Calcein-AM.

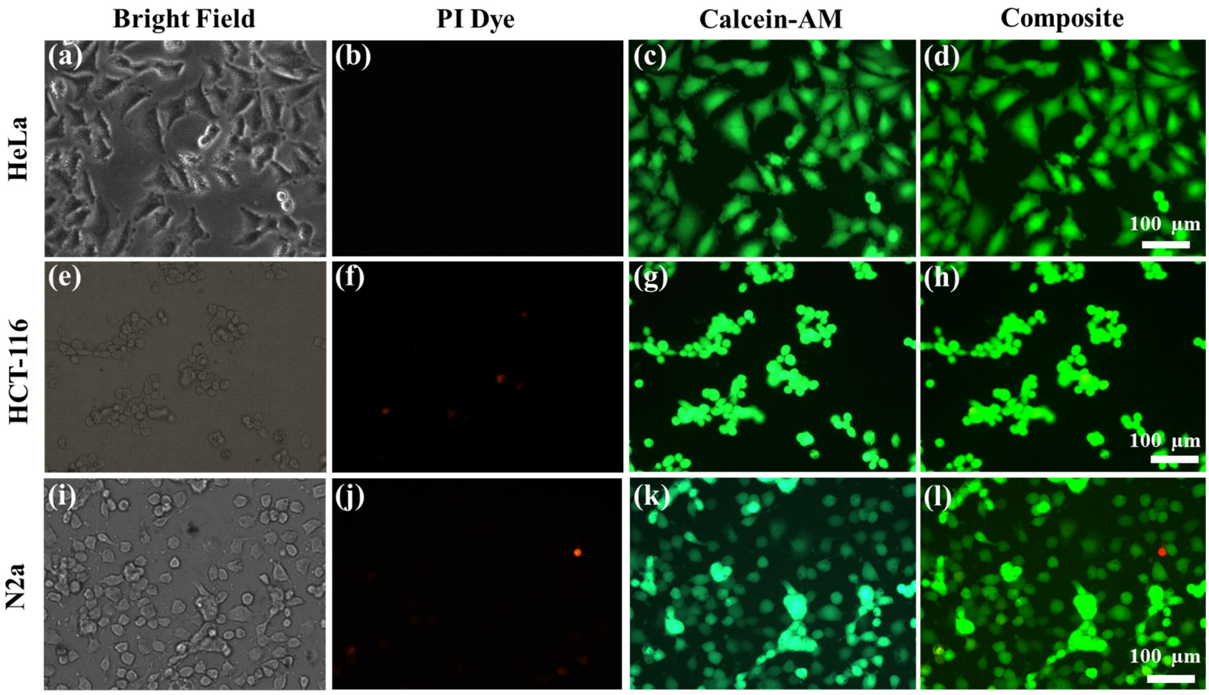

Figure S5. PI dye delivery and cell viability of HeLa, HCT-116, and N2a cells without Au-PNP attachment onto the cell surface after pulsed laser exposure (a,e,i) Bright-field image on laser exposure area without Au-PNP (b,f,j) No PI dye delivery without Au-PNP (c,g,k) Cell viability using Calcein-AM staining shows all cells are viable after laser exposure (d,h,l) Composite image of PI dye and Calcein-AM staining.

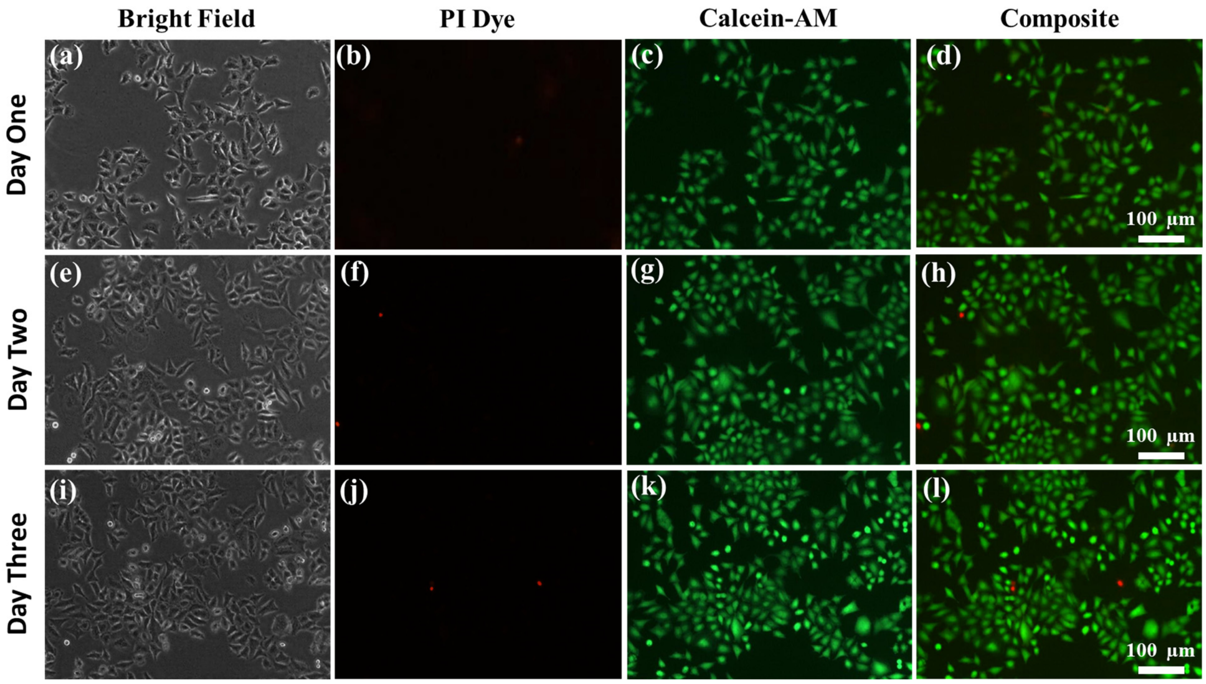

Figure S6. A control experiment using Au-PNP and pulse laser interaction of different days for HeLa cells. (a) Bright-field image using Au-PNP and laser interaction after one day (b) dead cell detection using PI dye. (c) Viability image using Calcein-AM staining. (d) Composite image of PI dye and Calcein-AM staining. (e,i) Bright-field image using Au-PNP and laser interaction after two days and three days. (f,j) Dead cell detection using PI dye. (g,k) Viability image using Calcein-AM staining. (h,l) Composite image of PI dye, and Calcein-AM staining.

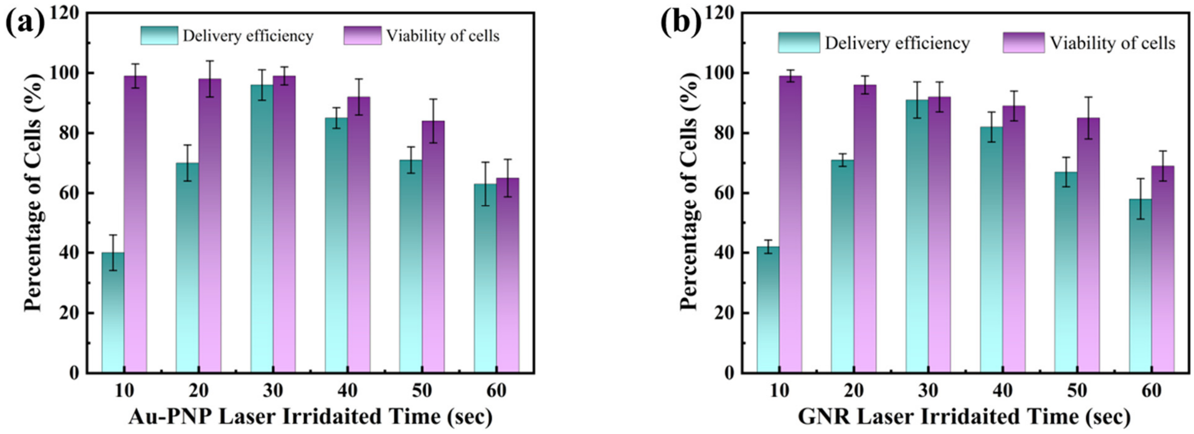

Figure S7. Time-dependent delivery efficiency and viability at 670 nm and 1030 nm for HeLa cells with Au-PNP and GNR respectively as a mediator. Due to the increase in time, the delivery efficiency increases and it is maximum at 30 seconds, and then the delivery efficiency and viability decrease with the increase of time. The laser fluence was 27 mJ/cm^2 with a 10 Hz pulse repetition rate. Data show average ± standard deviation (n =3 replicate).

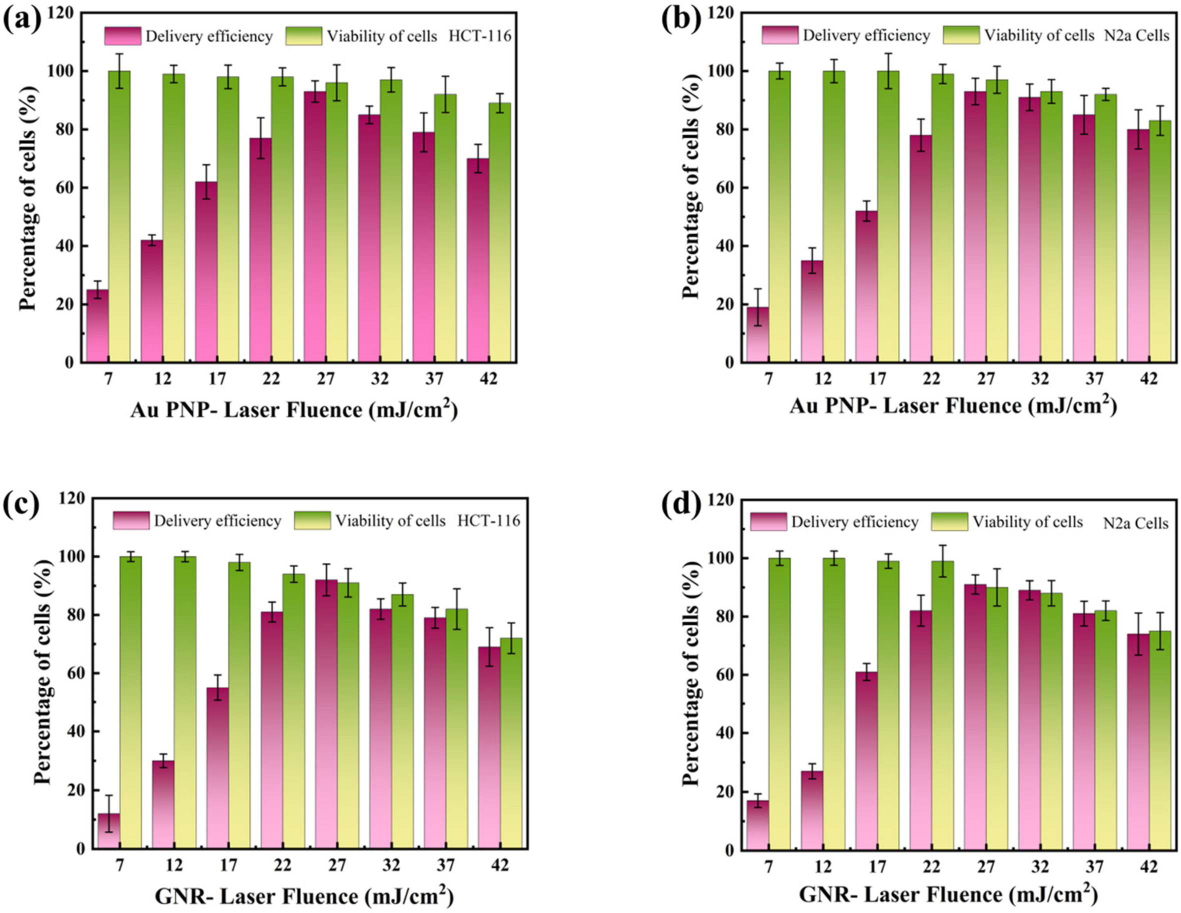

Figure S8. Illustrates the dynamic interplay between nanosecond pulse laser fluence and the intracellular delivery efficiency, as well as cell viability, in HCT-116 and N2a cells at wavelengths of 670 nm and 1030 nm. (a,b) delineate the impact of varying laser fluence on delivery efficiency and cell viability at 670 nm for HCT-116 and N2a cells, respectively. (c,d) portray the delivery efficiency and cell viability at 1030 nm for HCT-116 and N2a cells, respectively.

The data are presented as the average values with standard deviations (n=3 replicates).

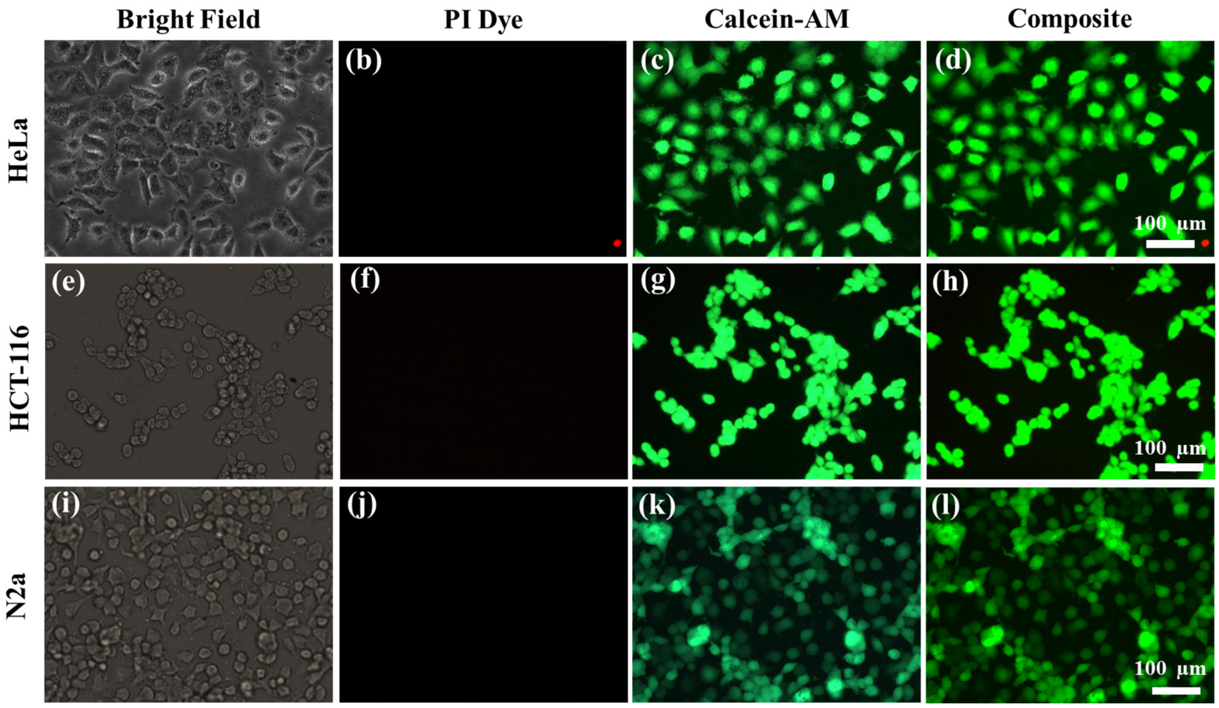

Figure S9. Showcases the delivery efficiency and viability assessment at a non-resonant wavelength of 600 nm, utilizing Au-PNP as a mediator, across HeLa, HCT-116, and N2a cell lines. (a, e, i) bright field images of HeLa, HCT-116, and N2a cells, respectively, following laser exposure. (b, f, j) depict red fluorescence, indicative of PI dye delivery (with no PI dye observed in cells). (c, g, k) green fluorescence signal, denoting maximally viable cells post-Calcein-AM staining, (d, h, l) provides a composite image overlaying PI dye and Calcein-AM staining image. Each experimental condition involved a 30-second laser exposure at a 10 Hz pulsing frequency, with a laser fluence of 27mJ/cm2.

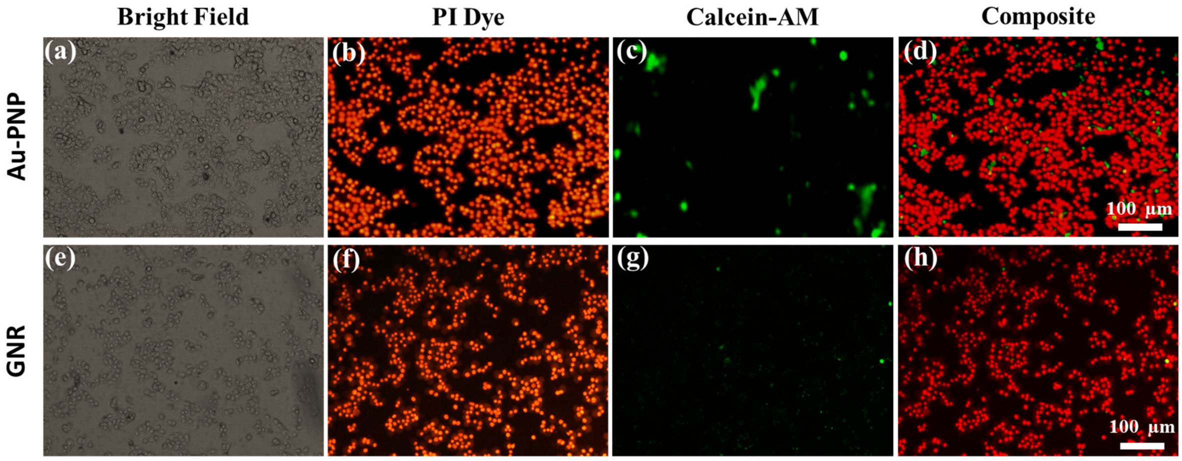

Figure S10. PI dye delivery at 670 nm and 1030 nm with Au-PNP and GNR respectively. (a-e) Bright-field image shows maximum cells are dead after laser exposure (b-f) Higher laser exposure, PI dye only stains all broken cytosol of dead cells (c-g) Cells viability test using Calcein-AM shows only 4-7 % cells are live (d-h) Composite image of PI dye and Calcein-AM staining. The laser exposure was 30 seconds at a 10 Hz pulsing frequency with 50 mJ/cm^2 laser fluence.

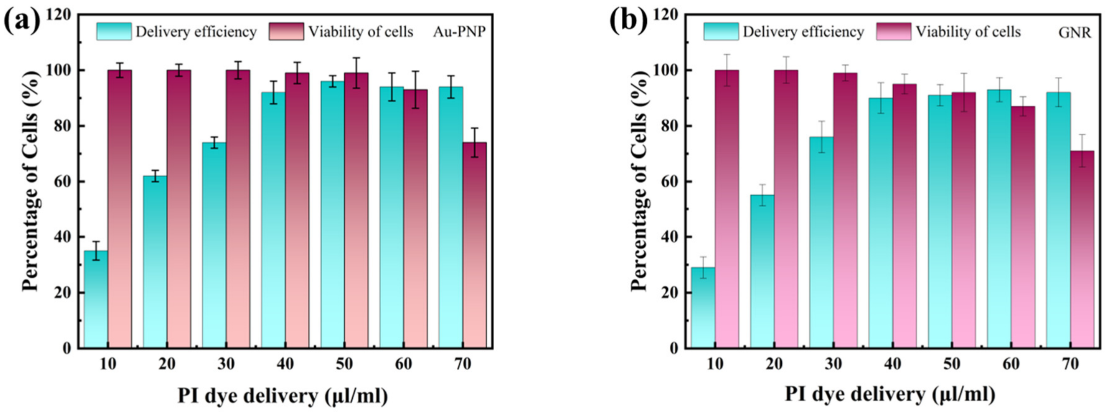

Figure S11. PI dye concentration dependent delivery efficiency and cell viability of HeLa cells at 670 nm and 1030 nm wavelength with 27 mJ/cm^2 laser fluence for 30 seconds.

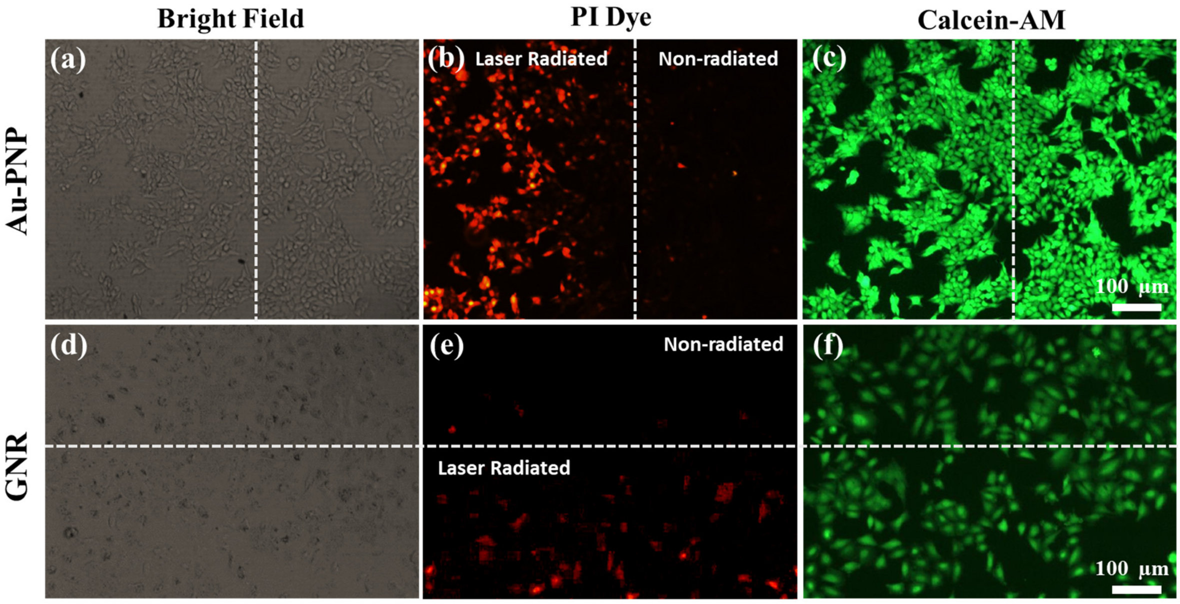

Figure S12. PI Dye delivery assessment on laser-radiated and non-radiated areas of HeLa Cells. (a,d) Bright-field image illustrating laser-radiated and non-radiated areas, distinctly separated by a white dotted line. (b,e) PI dye was selectively delivered to the laser-radiated region. (c,f) Cell viability was assessed using Calcein-AM staining after PI dye delivery, revealing viability across both radiated and non-radiated areas. The laser radiation was for 30 seconds at a 10 Hz pulsing frequency, utilizing a 27 mJ/cm^2 laser fluence at 670 nm and 1030 nm respectively.

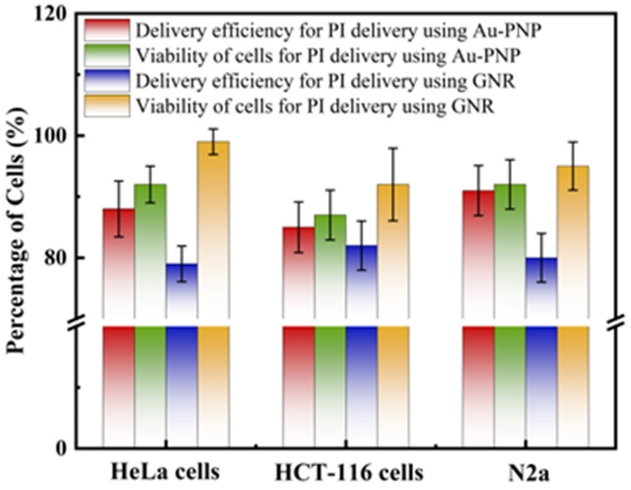

Figure S13 Delivery efficiency and cell viability at 538 nm and 560 nm wavelengths under Au-PNP and GNR mediation respectively. The laser exposure was 30 seconds at a 10 Hz pulsing frequency with 27mJ/cm^2 laser fluence. Data show average ± standard deviation (n =3 replicate).
